# Dietary Lecithin Decreases Skeletal Muscle COL1A1 and COL3A1 Gene Expression in Finisher Gilts

**DOI:** 10.3390/ani6060038

**Published:** 2016-06-07

**Authors:** Henny Akit, Cherie Collins, Fahri Fahri, Alex Hung, Daryl D’Souza, Brian Leury, Frank Dunshea

**Affiliations:** 1 Faculty of Veterinary and Agricultural Sciences, The University of Melbourne, Parkville, Victoria 3010, Australia; Fahri.Fahri@foodauthority.nsw.gov.au (F.F.); alex.hung@lucta.com (A.H.); brianjl@unimelb.edu.au (B.L.); fdunshea@unimelb.edu.au (F.D.); 2 Department of Animal Science, Faculty of Agriculture, Universiti Putra Malaysia, UPM Serdang, Selangor 43400, Malaysia; 3 Rivalea Australia Pty Ltd., Corowa 2646, Australia; ccollins@rivalea.com.au; 4 Australian Pork Ltd., Level 2, 2 Brisbane Ave, Barton Australian Capital Territory 2600, Australia; darryl.dsouza@australianpork.com.au

**Keywords:** collagen, gene expression, lecithin, muscle, finisher pig

## Abstract

**Simple Summary:**

In this study, the effect of dietary lecithin on skeletal muscle gene expression of collagen precursors and enzymes was investigated in gilts. Thirty-six finisher gilts were fed with diets containing either 0, 4, 20 or 80 g/kg soybean lecithin for six weeks. Then, rectus abdominis muscle was sampled and analyzed for eight genes involved in collagen synthesis and degradation (COL1A1, COL3A1, MMP-1, MMP-13, TIMP-1, TIMP-3, lysyl oxidase and α-subunit P4H) using quantitative real-time PCR. The results showed that lecithin down-regulated COL1A1 and COL3A1 as well as tended to down-regulate α-subunit P4H expression.

**Abstract:**

The purpose of this study was to investigate the effect of dietary lecithin on skeletal muscle gene expression of collagen precursors and enzymes involved in collagen synthesis and degradation. Finisher gilts with an average start weight of 55.9 ± 2.22 kg were fed diets containing either 0, 4, 20 or 80 g/kg soybean lecithin prior to harvest for six weeks and the rectus abdominis muscle gene expression profile was analyzed by quantitative real-time PCR. Lecithin treatment down-regulated Type I (α1) procollagen (COL1A1) and Type III (α1) procollagen (COL3A1) mRNA expression (*p* < 0.05, respectively), indicating a decrease in the precursors for collagen synthesis. The α-subunit of prolyl 4-hydroxylase (P4H) mRNA expression also tended to be down-regulated (*p* = 0.056), indicating a decrease in collagen synthesis. Decreased matrix metalloproteinase-1 (MMP-1) mRNA expression may reflect a positive regulatory response to the reduced collagen synthesis in muscle from the pigs fed lecithin (*p* = 0.035). Lecithin had no effect on tissue inhibitor metalloproteinase-1 (TIMP-1), matrix metalloproteinase-13 (MMP-13) and lysyl oxidase mRNA expression. In conclusion, lecithin down-regulated COL1A1 and COL3A1 as well as tended to down-regulate α-subunit P4H expression. However, determination of muscle collagen content and solubility are required to support the gene functions.

## 1. Introduction

Soy lecithin is a by-product from the processing of soybean oil. It is widely used in the food industry as an emulsifier and stabilizing agent in the production of foods such as margarine, mayonnaise, chocolate and baked goods. Lecithin is also consumed because of its purported health benefits. For example, a review [1] reported numerous data supporting the effect of lecithin in lowering the blood cholesterol level in hyperlipidemic animals and humans. The prevention and treatment of alcoholic liver disease have also provided evidence that components of lecithin have anti-fibrogenic properties. Lecithin not only prevented hepatic collagen accumulation through decreased collagen synthesis [2,3,4], but also enhanced the breakdown of existing collagen [5] through stimulation of collagenase activity [6]. Regulation of collagen synthesis could be exerted at the gene level, during transcription and translation levels within the cells, as well as during the extracellular assembly and cross-linking [7]. The determination of RNA expression of specific collagen types and prolyl-4 hydroxylase activity levels has been used to investigate collagen synthesis [8]. Dilinoleoylphosphatidylcholine (DLPC), an active ingredient of lecithin, down-regulated Type I (α1) procollagen (COL1A1) gene expression [2,3,4] in hepatic cells. The latter authors suggested that the decreased collagen molecule precursor gene expression and collagen synthesis by lecithin may explain the mechanisms involved in the prevention of collagen accumulation in cases of alcoholic liver fibrosis. However, to the best of our knowledge, there are no published articles that have investigated the effect of lecithin on key genes involved in intramuscular collagen synthesis and degradation. Therefore, the aim of this study was to investigate the effect of dietary lecithin on muscle collagen precursors COL1A1 and Type III (α1) procollagen (COL3A1), α-subunit of prolyl 4-hydroxylase (P4H), and lysyl oxidase as well as matrix metalloproteinase-1 (MMP-1), matrix metalloproteinase-13 (MMP-13), tissue inhibitor metalloproteinase-1 (TIMP-1) and tissue inhibitor metalloproteinase-3 (TIMP-3) mRNA expression levels. 

## 2. Materials and Methods 

### 2.1. Animals and Experimental Design

The experiment was conducted at Rivalea (Australia), Corowa, NSW and all procedures outlined in this investigation were approved by the Rivalea Animal Care and Ethics Committee (09M073C). Thirty-six Large White × Landrace crossbred finisher gilts (PrimeGro^TM^ Genetics, Rivalea Pty Ltd., Corowa, NSW, Australia) were randomly allocated into individual pens at entry to the facility at 15 weeks old with an average weight of 55.9 ± 2.22 kg. During a one-week acclimatization period, the pigs were fed the control diet (commercial finisher diet) at 80% of *ad libitum*. After the acclimatization period, the pigs were split into three blocks (each containing 12 pigs) and started on the test diets over three days. Blocking factor was the start date for test diets. Each block was started on the test diets on different days to ensure a smooth transfer of the pigs from the farm to the abattoir for slaughter and carcass data collection over three days. The test diets were randomly allocated within each of the blocks that included (i) control diet; (ii) Ultralec^®^ F de-oiled soybean lecithin (ADM Australia Pty Ltd., Bondi Junction, NSW, Australia) at 4 g/kg of commercial finisher diet; (iii) soybean lecithin at 20 g/kg of commercial finisher diet; and (iv) soybean lecithin at 80 g/kg of commercial finisher diet. All diets were formulated to contain 0.6 g available lysine/megajoule (MJ) digestible energy (DE) and 14.2 MJ DE/kg. Diets were pelleted and fed to the pigs starting from 17 weeks old through to slaughter at 23 weeks old. All pigs had *ad libitum* access to feed and water via nipple drinkers for six weeks prior to reaching an average final slaughter live weight of 103.9 ± 6.40 kg. The pigs were slaughtered in a commercial abattoir at the conclusion of the 42 days experimental period. The pigs were stunned using a carbon dioxide Dip-lift stunner (Butina^®^, Sjelland, Denmark) set at 85% CO_2_ and exposed for 1.8 min. Exsanguination, scalding, de-hairing and evisceration were performed according to the standard procedures practiced in commercial abattoirs. The rectus abdominis muscles were collected 25 min post-slaughter in Falcon^®^ centrifuge tubes (Thermo Fisher Scientific Inc., Waltham, MA, USA) and immediately immersed in liquid nitrogen, then stored at −80 °C. This muscle was selected because of convenience of sampling at the slaughter house.

### 2.2. RNA Extraction and cDNA Synthesis

Approximately 200–300 mg of the muscle powder was pulverized in the presence of liquid nitrogen using a mortar and pestle. The total RNA was extracted using Trizol^®^ reagent (Invitrogen, San Diego, CA, USA) followed by isolation using PureLink^TM^ Micro-to-Midi RNA isolation system kit (Invitrogen, San Diego, CA, USA) following instructions of the manufacturer. The yield and integrity of the extracted RNA were determined using Experion^TM^ automated electrophoresis system (BIORAD, Gladesville, NSW, Australia) and Experion^TM^ RNA StdSens analysis kit (BIORAD, Gladesville, NSW, Australia). Samples displaying RNA quality indicator (RQI) ≥ 8.0 and 28S:18S ribosomal RNA ratio close to 1.5 were considered sufficient quality RNA. One microgram total RNA from each sample was reverse transcribed into cDNA using the SuperScript^®^ III First-Strand Synthesis System (Invitrogen, San Diego, CA, USA) for RT-PCR and 50 ng/μL random hexamer primer (Invitrogen, San Diego, CA, USA), according to manufacturer’s instructions into a final volume of 20 μL and stored at −80 °C. 

### 2.3. Primer Design

The sequences of genes of interest were obtained from the National Centre for Biotechnology Information database [9]. The genes sequences were copied into the Invitrogen OligoPerfect^TM^ Designer software (Invitrogen, San Diego, CA, USA) [10] and this software locates primer sequences within the given mRNA sequence that meets individual specifications of size, annealing temperature, Guanine and Cytosine (GC) content, region of analysis, product size, salt concentration and primer concentration. Then, the primers for the genes were designed using Netprimer (PREMIER Biosoft International, Palo Alto, CA, USA) [11]. Only primers MMP-1 and Ribosomal 18s (R18s) were designed based on porcine sequences. As complete genomes of the pig are currently only partially sequenced, COL1A1, COL3A1, α-subunit of P4H, lysyl oxidase, MMP-13, TIMP-1 and TIMP-3 primers were designed based on mouse (*Mus musculus*) sequences. Characteristics of the primers used for real-time quantitative PCR are listed in Table 1. R18s was used as the reference gene for normalization because its expression level was constant across all test samples and was not affected by the experimental treatment compared to β-actin (data not shown). The primers were validated using a real-time PCR of cDNA synthesized from an RNA pool made of all samples. A standard curve was generated from serial dilutions of cDNA by plotting the log of the starting dilution factor of the template against cycle threshold (CT) value obtained during amplification of each dilution. The amplification efficiency (E) was calculated using the formula E = (10^−1/slope^ − 1) × 100. The amplification efficiency for all tested genes varied from 95% to 100% (Table 2). All reactions were done in triplicate and the coefficient of determination (R^2^) of all standard curves were >0.90, indicating acceptable accuracy and reproducibility (Table 2). The specificity of primers was confirmed by melting curve analysis. A non-template (without cDNA) reaction was included with each PCR run to validate that primers were not amplifying contaminating DNA.

### 2.4. Quantitative Real-Time PCR

The cDNA were amplified by real-time PCR using SYBR^®^ Green I (BIORAD, Gladesville, NSW, Australia) on the BIORAD MyiQ^TM^ Single-Colour Real-Time PCR Detection System (BIORAD, Gladesville, NSW, Australia) following the manufacturer’s instructions. Each PCR reaction contained 2 μL of cDNA mixed with 1–3 μL of each primer (volume fluctuates depending on the optimized primer concentrations as shown in Table 2), 12.5 μL of SYBR^®^ Green I Supermix (BIORAD, Gladesville, NSW, Australia) and RNase free water (Invitrogen, San Diego, CA, USA) to make up a final volume of 25 μL. The thermo cycle protocol consisted of initial hot start at 95 °C for 3 min as the initial denaturation step of one cycle, followed by 40 cycles at 95 °C for 10 s (denaturation), 51.0 to 61.2 °C for 45 s (annealing) and an extension step at 95.0 °C for 1 min. Optimized annealing temperatures for each gene are specified in Table 2. PCR runs for each sample were performed in triplicate. The CT values for each target gene were normalized with the reference gene (R18s) for both test and the control groups. The mean ΔCT value was used in the ΔCT method to calculate the relative expression values for the test and the control groups. The fold change in each target gene mRNA level in the lecithin diet was calculated relative to the control diet which was set to 1.

Reverse

GACTCAACACGGGAAACCTC

### 2.5. Statistical Analysis

Data were analyzed using a single-factor analysis of variance (ANOVA) with a fixed effect model for dietary treatment using GenStat (13 Ed., VSN International Ltd., Hemel Hempstead, Dacorum, UK). The contrasts assessed were for control *versus* pooled lecithin treatments as there were no dose effects (data not shown). The results were considered statistically significant when *p* < 0.05 and were considered as trends when 0.05 ≤ *p* ≤ 0.10. The TIMP-3 gene had a ΔCT value of 50.5 which was considered relatively high (data not shown). This may indicate low amplification and so these data have not been reported.

## 3. Results

**Figure 1:**
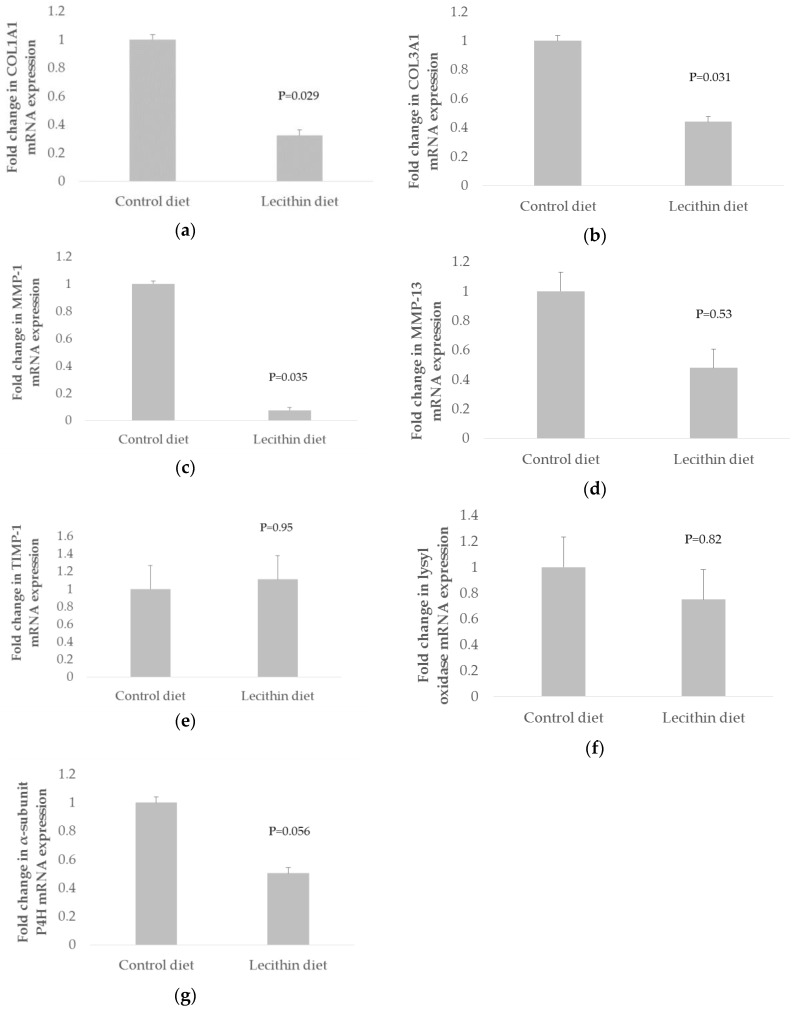
Effects of dietary lecithin on (**a**) Type I (α1) procollagen (COL1A1); (**b**) Type III (α1) procollagen (COL3A1); (**c**) matrix metalloproteinase-1 (MMP-1); (**d**) matrix metalloproteinase-13 (MMP-13); (**e**) tissue inhibitor metalloproteinase-1 (TIMP-1); (**f**) lysyl oxidase; and (**g**) α-subunit of prolyl 4-hydroxylase (α-subunit P4H) gene expression. mRNA levels were measured in triplicate in each rectus abdominis muscle from pigs fed with control diet (0 g/kg lecithin) or lecithin diet that were pooled across doses of lecithin (4, 20 and 80 g/kg lecithin). The expression level normalized against the Ribosomal 18s (R18s) reference gene. Fold change in COL1A1, COL3A1, α-subunit of P4H, lysyl oxidase, MMP-1, MMP-13 and TIMP-1 mRNA levels in lecithin diet was calculated relative to the control diet which was set as 1. Error bars indicate standard error (SE) of control (*n* = 9) or lecithin (*n* = 27) diet group. Mean values statistically significantly different from control in pair comparison (*p* < 0.05 in ANOVA). The results were considered as trends when 0.05 ≤ *p* ≤ 0.10 in ANOVA.

Pigs fed dietary lecithin for six weeks significantly (*p* < 0.05) down-regulated skeletal muscle COL1A1, COL3A1 and MMP-1 mRNA expression by 0.33-fold, 0.44-fold and 0.08-fold, respectively, compared to pigs fed with the control diet (Figure 1a–c). Dietary lecithin tended (*p* = 0.056) to down-regulate α-subunit P4H mRNA expression by 0.5-fold, compared to pigs fed with the control diet (Figure 1g). Dietary lecithin had no significant effect on MMP-13, TIMP-1 and lysyl oxidase mRNA expression, when compared to pigs fed with the control diet (*p* > 0.05) (Figure 1d–f).

## 4. Discussion

In the present study, we report for the first time that dietary lecithin decreased mRNA levels of COL1A1 and COL3A1 in the skeletal muscle of pigs. Fibrillar collagen Types I and III are involved in collagen fibril organization and collagen biosynthesis. The synthesis rates of Types I and III collagen are regulated by their corresponding mRNA expression levels [12]. Hence, the down-regulation of COL1A1 and COL3A1 expression in the present study may indicate a reduction in the synthesis rates of Types I and III collagen, respectively. The fibrillar collagen Types I and III mRNA expression were well coordinated with α-subunit P4H mRNA expression and prolyl-4 hydroxylase activity in rat skeletal muscle induced with a mechanical stimulus [13,14]. A simultaneous decrease in collagen precursors and α-subunit P4H mRNA expression were evident in the present experiment, although enzyme activity was not measured. The level of α-subunit P4H mRNA expression is an important determinant in the initiation of collagen biosynthesis [15] and its reduced level of expression reflects improper formation of a functional collagen fibril.

The effect of DLPC has been studied extensively on liver fibrosis treatment and prevention caused by alcohol abuse. DLPC prevented collagen accumulation as evidenced by down-regulation of COL1A1 expression in hepatocyte cell culture [2,3,4]. Dietary lecithin supplement in pigs reduced chewiness and hardness of the longissimus muscle [16] and the improvement in pork chewiness was suggested to be associated with reduced intramuscular collagen [17]. As a point of reference, the collagen content of the longissimus muscle was 1.51, 1.13, 1.09 and 1.07 mg/g for these same pigs fed 0, 4, 20 and 80 g/kg dietary lecithin, respectively [17]. Since COL1A1 and COL3A1 are precursors for collagen synthesis, it is necessary to investigate if lecithin could have a similar effect in reducing the collagen content of the rectus abdominis muscle to support the gene functions. Collagen solubility is the main basis for the determination of intramuscular collagen contribution to meat toughness [18,19] and increased collagen solubility positively correlated with tenderness [20,21]. Collagen type was shown to contribute to meat collagen solubility. For example, Bao *et al.* [22] reported a negative correlation between muscle COL3A1 expression and collagen solubility. Similarly, Burson *et al.* [23] found a negative correlation between the muscle percentage of Type III collagen and collagen solubility (*r* = −0.49) in beef. It is, however, necessary to investigate the effect of lecithin on collagen solubility if its relationship to meat tenderness is to be studied. 

Our study showed that lecithin decreased MMP-1 expression which may reflect a positive regulatory response to the reduced collagen synthesis. MMP-1 and MMP-13 were identified to being involved in the degradation of fibrillar collagen Types I and III [24,25,26]. Once the MMPs are present in active forms in the extracellular matrix, the tissue inhibitor metallo proteinases (TIMPs) inhibit active MMPs by binding in a 1:1 molar ratio to establish a balance between collagen synthesis and degradation. 

## 5. Conclusions

In conclusion, supplemental lecithin down-regulated expression of the key genes involved in collagen synthesis, namely COL1A1 and COL3A1, and it tended to down-regulate the expression of α-subunit P4H. As a result of this, it may be possible that the MMP-1 expression was down-regulated to control any further decrease of collagen in the muscle of pigs fed lecithin.

## Figures and Tables

**Table 1:** Characteristics of the primers used for quantitative real-time PCR.

Gene ^1^	Species	Primers	Primer Sequence (from 5′–3′)	Accession Number	Amplicon Size (bp)
COL1A1	Mouse	Forward	GTCTGGTTTGGAGAGAGCAT	BC050014.1	189
		Reverse	CTTCTTGAGGTTGCCAGTCT		
COL3A1	Mouse	Forward	TGATGTCAAGTCTGGAGTGG	NM_009930.2	223
		Reverse	TCCTGACTCTCCATCCTTTC		
MMP-1	Pig	Forward	GTTCCACAAATGAGTGCTGA	EU722905.1	212
		Reverse	ATAATAACGACGGCTCATCC		
MMP-13	Mouse	Forward	GTGACTGGCAAACTTGATGA	BC125320.1	211
		Reverse	TCACATCAGACCAGACCTTG		
TIMP-1	Mouse	Forward	CCCAGAAATCAACGAGA	BC034260.1	154
		Reverse	TGGGACTTGTGGGCATA		
TIMP-3	Mouse	Forward	ACACGGAAGCCTCTGAAA	BC014713.1	231
		Reverse	TGGAGGTCACAAAACAAGG		
Lysyl oxidase	Mouse	Forward	CTGCTTGATGCCAACACA	M65142.1	156
		Reverse	TGCCGCATAGGTGTCATA		
α-subunit P4H	Mouse	Forward	CCCAGTCAGGTCTGCTATTC	BC009654.1	204
		Reverse	GGAACAGTCTCTGGACAACC		
R18s	Pig	Forward	GAACGCCACTTGTCCCTCTA	AY265350.1	219
		Reverse	GACTCAACACGGGAAACCTC		

^1^ COL1A1 = Type I (α1) procollagen; COL3A1 = Type III (α1) procollagen; MMP-1= Matrix metalloproteinase-1; MMP-13 = Matrix metalloproteinase-13; TIMP-1 = Tissue inhibitor metalloproteinase-1; TIMP-3 = Tissue inhibitor metalloproteinase-3; α-subunit P4H = α-subunit of prolyl 4-hydroxylase; R18s = Ribosomal 18 s.

**Table 2:** Optimized quantitative real-time PCR conditions for genes of interest.

Gene ^1^	Optimized Annealing Temperature (°C)	Optimized Primer Concentration (nM)	Optimized Threshold Cycle (CT)	Coefficient of Determination (R^2^)	Amplification Efficiency (%)
COL1A1	60.9	50	310.00	0.903	96.4
COL3A1	53.4	200	128.80	0.976	100
MMP-1	60.9	100	335.42	0.963	99.8
MMP-13	58.7	700	310.65	0.929	95.2
TIMP-1	58.7	1100	189.70	0.924	99.9
TIMP-3	60.9	300	125.02	0.994	99.9
Lysyl oxidase	58.7	200	195.72	0.952	100
α-subunit P4H	51.0	400	280.84	0.923	98.0
R18s	61.2	60	201.50	0.999	97.4

^1^ COL1A1 = Type I (α1) procollagen; COL3A1 = Type III (α1) procollagen; MMP-1= Matrix metalloproteinase-1; MMP-13 = Matrix metalloproteinase-13; TIMP-1 = Tissue inhibitor metalloproteinase-1; TIMP-3 = Tissue inhibitor metalloproteinase-3; α-subunit P4H = α-subunit of prolyl 4-hydroxylase; R18 s = Ribosomal 18 s.
